# Exploring the Foundation of Genomics: A Northern Blot Reference set for the Comparative Analysis of Transcript Profiling Technologies

**DOI:** 10.1002/cfg.443

**Published:** 2004-12

**Authors:** Danielle Kemmer, Margareta Faxén, Emily Hodges, Jonathan Lim, Elena Herzog, Elsebrit Ljungström, Anders Lundmark, Mary K. Olsen, Raf Podowski, Erik L. L. Sonnhammer, Peter Nilsson, Mark Reimers, Boris Lenhard, Steven L. Roberds, Claes Wahlestedt, Christer Höög, Pankaj Agarwal, Wyeth W. Wasserman

**Affiliations:** 1 Center for Genomics and Bioinformatics Karolinska Institutet Stockholm Sweden; 2 Centre for Molecular Medicine and Therapeutics Department of Medical Genetics University of British Columbia 950 West 28th Avenue Vancouver BC V5Z 4H4 Canada; 3 CNS Genomics Unit, Pharmacia Corporation 301 Henrietta Street Kalamazoo Michigan 49007 USA; 4 Royal Institute of Technology Department of Biotechnology Division of Molecular Biotechnology Stockholm 106 91 Sweden; 5 Bioinformatics Group GlaxoSmithKline King of Prussia PA USA; 6 National Cancer Institute Bethesda MD USA; 7 Pfizer Research and Development Chesterfield MO USA

## Abstract

In this paper we aim to create a reference data collection of Northern blot results and demonstrate how such a collection can enable a quantitative comparison of modern expression profiling techniques, a central component of functional genomics studies. Historically, Northern blots were the *de facto* standard for determining RNA transcript levels. However, driven by the demand for analysis of large sets of genes in parallel, high-throughput methods, such as microarrays, dominate modern profiling efforts. To facilitate assessment of these methods, in comparison to Northern blots, we created a database of published Northern results obtained with a standardized commercial multiple tissue blot (dbMTN). In order to demonstrate the utility of the dbMTN collection for technology comparison, we also generated expression profiles for genes across a set of human tissues, using multiple profiling techniques. No method produced profiles that were strongly correlated with the Northern blot data. The highest correlations to the Northern blot data were determined with microarrays for the subset of genes observed to be specifically expressed in a single tissue in the Northern analyses. The database and expression profiling data are available via the project website (http://www.cisreg.ca). We believe that emphasis on multitechnique validation of expression profiles is justified, as the correlation results between platforms are not encouraging on the whole. Supplementary material for this article can be found at: http://www.interscience.wiley.com/jpages/1531-6912/suppmat


**Comparative and Functional Genomics**
*Comp Funct Genom* 2004; **5**: 584–595.
Published online in Wiley InterScience (www.interscience.wiley.com). **DOI:** 10.1002/cfg.443

**Research Paper**

### Exploring the foundation of genomics: a Northern blot reference set for the comparative analysis of transcript profiling technologies

Danielle Kemmer[1#], Margareta Faxén[1#], Emily Hodges[1], Jonathan Lim[2], Elena Herzog[1], Elsebrit Ljungström[1], Anders Lundmark[1], Mary K. Olsen[3], Raf Podowski[1], Erik L. L. Sonnhammer[1], Peter Nilsson[4], Mark Reimers[1##], Boris Lenhard[1], Steven L. Roberds[3###], Claes Wahlestedt[1], Christer Höög[1], Pankaj Agarwal[5] and Wyeth W. Wasserman[2*]

[1]*Center for Genomics and Bioinformatics, Karolinska Institutet, Stockholm, Sweden*
[2]*Centre for Molecular Medicine and Therapeutics, Department of Medical Genetics, University of British Columbia, Vancouver, BC, Canada*
[3]*CNS Genomics Unit, Pharmacia Corporation, 301 Henrietta Street, Kalamazoo, Michigan 49007, USA*
[4]*Royal Institute of Technology, Department of Biotechnology, Division of Molecular Biotechnology, 106 91 Stockholm, Sweden*
[5]*Bioinformatics Group, GlaxoSmithKline, King of Prussia, PA, USA*

**Correspondence to: Wyeth W. Wasserman, Centre for Molecular Medicine and Therapeutics, University of British Columbia, 950 West 28th Avenue, Vancouver BC V5Z 4H4, Canada. E-mail: wyeth@cmmt.ubc.ca*

*[#]These authors contributed equally to this work.*

*[##]Present address: National Cancer Institute, Bethesda, MD, USA.*

*[###]Present address: Pfizer Research and Development, Chesterfield, MO, USA.*

Accepted: 19 November 2004

### Abstract

**In this paper we aim to create a reference data collection of Northern blot results and demonstrate how such a collection can enable a quantitative comparison of modern expression profiling techniques, a central component of functional genomics studies. Historically, Northern blots were the *de facto* standard for determining RNA transcript levels. However, driven by the demand for analysis of large sets of genes in parallel, high-throughput methods, such as microarrays, dominate modern profiling efforts. To facilitate assessment of these methods, in comparison to Northern blots, we created a database of published Northern results obtained with a standardized commercial multiple tissue blot (dbMTN). In order to demonstrate the utility of the dbMTN collection for technology comparison, we also generated expression profiles for genes across a set of human tissues, using multiple profiling techniques. No method produced profiles that were strongly correlated with the Northern blot data. The highest correlations to the Northern blot data were determined with microarrays for the subset of genes observed to be specifically expressed in a single tissue in the Northern analyses. The database and expression profiling data are available via the project website (http://www.cisreg.ca). We believe that emphasis on multi-technique validation of expression profiles is justified, as the correlation results between platforms are not encouraging on the whole. Supplementary material for this article can be found at: http://www.interscience.wiley.com/jpages/1531-6912/suppmat**
**Copyright © 2005 John Wiley & Sons, Ltd.**

**Keywords:** gene expression; Northern; microarray; genomics; database

### Introduction

Technologies to monitor gene expression are abundant, and have been widely applied to characterize genes and to analyse expression at a genome scale [1,2]. Most approaches are based on the determination of mRNA abundance, which serves as a first approximation for the strength of a gene's expression in a cell or tissue sample. Despite this common basic principle of expression profiling techniques, each exhibits distinct strengths

Copyright © 2005 John Wiley & Sons, Ltd.

and weaknesses that render certain techniques preferable, depending on the scientific goal. Technically, the diverse methods for transcript profiling can be broadly categorized into three distinct sets: (a) hybridization-based; (b) sequencing-based; and (c) PCR-based.

Historically, transcript levels of newly cloned genes have been assessed primarily with Northern blots, which remain a popular but nonetheless labour-intensive hybridization-based technique for the analysis of individual genes. For the study of large sets of genes required in genomics, transcript levels are often monitored by array-based hybridization methods. Several variations on high-throughput arrays have been developed, including cDNA macroarrays on nylon filters, cDNA microarrays on glass, and oligonucleotide arrays [3–5]. Despite their popularity, questions remain about the capacity of array-based methods to assess accurately the level of gene expression in terms of linearity between signal and expression [6].

Sequencing-based methods measure transcript frequency within cDNA or SAGE libraries [7,8]. These highly comprehensive approaches allow the detection of unexpected transcripts and therefore make valuable contributions to gene discovery [9]. However, a major drawback for the most accessible tag data, the analysis of EST sequences, are normalization procedures used in the construction of many cDNA libraries, which result in non-quantitative data.

PCR-based approaches are used extensively for expression profiling of small sets of genes. RT-PCR is a sensitive and powerful tool for the semi-quantitative analysis of relative transcript levels [10]. Quantitative approaches, such as TaqMan®, have been developed for detailed studies of single genes [11], but high-throughput analysis is prohibitively expensive in terms of both labour and reagents.

Given the plethora of competing profiling methods available to researchers, it is essential to determine their respective merits and faults by comparison to standard sets of gene expression profiles. To date, there have been a limited number of pairwise comparisons of expression profiling technologies [1,2,4,12–14], but no broad cross-platform studies have been reported. A significant requirement for conducting multi-platform comparisons is a suitable reference collection. For newly cloned human genes, a *de facto* standard for expression profiling has emerged — multiple tissue Northern blots. In fact, most reports specifically characterizing a novel gene include a figure with a common format of multiple tissue Northern blots generated by a single commercial supplier (ClonTech). Thus, within the scientific literature there exists a large collection of peer-reviewed reference data describing the expression of human genes.

We report the creation of a database of published multiple tissue Northern blot results and demonstrate how such a database can facilitate comparison of expression profiles generated with diverse experimental platforms. First, we describe the procedures used to extract the published results from the literature, including the identification of articles, the densitometry of blot images, and the format of the data collection (dbMTN). By using RNA from the same commercial source, we were able to generate expression profiles with multiple techniques for comparison to the reference Northern data. We show the procedures and generate correlation scores describing the similarity between the profiles obtained with the different methods. The Northern blot reference collection, as well as our collection of profiles and protocols from diverse methods, are available for further analysis via an in-depth website.

### Materials and methods

### Database of results from ClonTech multiple tissue Northern blots

A database of expression profiles produced from Northern blots has been collected from publications utilizing common commercial multiple tissue filters. A curated list of articles containing MTN Northern blots (ClonTech) was obtained from the manufacturer. Each blot contains mRNA recovered from eight human tissues. With permission from the publishers, images were downloaded from the three journals with the greatest number of MTN-containing papers. These included *Genomics* (547 blots for 221 genes), *Journal of Biological Chemistry* (693 blots for 265 genes) and *Proceedings of the National Academy of Sciences of the USA* (155 blots for 67 genes). Images were analysed using the Gel-Pro Plus package (Media Cybernetics). A relative pattern of expression for each band

Copyright © 2005 John Wiley & Sons, Ltd.

(specific transcript in a single tissue) was generated by subtracting the highest density observed in band-free lanes and the vector was normalized to unit length. All data were reviewed to confirm that the recorded patterns of expression were consistent with the observed bands on the blots, and each transcript was annotated with an official identifier to facilitate future analysis.

### Oligonucleotides

PCR primers were designed using the MEDUSA program [21]. Gene-specific primer pairs preferentially flanked introns or overlapped splice junctions to decrease the likelihood of obtaining RT-PCR products from genomic DNA. HPLC-purified oligonucleotides were purchased from Interactiva Biotechnologie GmbH.

### RNA

Five tissues were selected for analysis: heart, brain, lung, liver, and skeletal muscle. To ensure uniformity, all RNA samples were purchased from ClonTech. The commercial preparations were generated from pools of tissue samples from multiple individuals. Total RNA for RT-PCR was treated with DNA Free (Ambion) to eliminate residual genomic DNA. The Northern blots obtained from several years of biological literature were generated with different pools of RNA isolated with the same production process.

### Analysis of nucleic acid preparations

A BioAnalyser 2100 (Agilent Technologies) was employed for quality control of total and poly $A^+$ RNA and for the analysis of RT-PCR products. RNA samples were loaded onto 'RNA chips' (RNA 6000 kit, Agilent) and analysed. In addition to the determination of both molecular size and concentration for defined bands, the analysis provides measures for RNA degradation and contamination by either genomic DNA or ribosomal RNA. DNA samples, e.g. PCR products for spotting onto arrays, were analysed with the DNA 500 assay (Agilent). Results acquired from these assays provide an accurate and consistent depiction of the molecular weight of observed bands, from which we were able to determine density ratios of background (alternative) bands to the expected product for each sample.

### RT-PCR

Total RNA was reverse transcribed in the presence of an oligo$(dT)_{20}$ primer, using avian RNase H-minus reverse transcriptase (ThermoScript RT-PCR System, Life Technologies). PCR reactions were performed on single-stranded cDNA in the presence of specific primer pairs. Reactions (25 µl) included AmpliTaq Gold™ DNA polymerase with the corresponding GeneAmp® 10× PCR Buffer (PE Biosystems) and a $MgCl_2$ concentration of 2.3 mM. The cycle settings were as follows: 95 °C for 10 min, 33 cycles of 95 °C for 15 s, 60 °C for 30 s and 72 °C for 45 s. At the conclusion, a final extension was performed at 72 °C for 7 min. PCR products were separated on 2% agarose gels.

### Amplification of cDNA for filter and cDNA array spotting

Two pools containing total RNA from human fetal brain and human testis or HeLa cells and human placenta were reverse transcribed under the conditions described above. PCR reactions (50 µl) were performed with the above conditions over 42 cycles. PCR products were purified using the QIAquick PCR Purification Kit (Qiagen) and analysed on the BioAnalyser.

### Filter macroarrays

### Array construction

0.5 µl denatured PCR products containing 5 ng DNA were printed in duplicate onto positively charged nylon membranes (Roche), using a robotic dispenser (Hydra, Robbins Scientific). The DNA was cross-linked to the membranes (Stratalinker, Stratagene).

### Probe synthesis

Complex probes were labelled with $[\alpha^{32}P]$-dCTP, using a reverse transcription reaction (SuperScript™, Life Technologies). Methods for simultaneous labelling and first strand cDNA synthesis were performed according to the following protocol. 1 µg mRNA in the presence of oligo$(dT)_{18}$ was heated to 70 °C for 5 min and cooled on ice. Next, the mixture was incubated at 42 °C for 1 h in the presence of 50 mM Tris–HCl, 75 mM KCl, 3 mM $MgCl_2$, 10 mM DTT, 500 µM each dATP, dGTP,

Copyright © 2005 John Wiley & Sons, Ltd. 

dTTP, 20 μM dCTP, 50 μCi [$\alpha^{32}$P]-dCTP and 200 U SuperScript II reverse transcriptase. After 1 h, reactions were terminated at 70 °C for 15 min. For RNA removal, reactions were incubated with 2 U RNase H at 37 °C for 20 min. Unincorporated nucleotides were removed by filtration through Sephadex G50 columns (Amersham Pharmacia Biotech). Specific activity was determined to be $2 \times 10^7$cpm/μl for each probe.

#### Hybridization

Prior to hybridization, membranes were rinsed in 2× SSC at room temperature and pre-hybridized with 10 ml PerfectHyb (Sigma) for 1 h at 65 °C. Labelled probes were denatured at 95 °C for 5 min and cooled on ice. Probes were mixed with 5 ml hybridization solution and incubated with membranes overnight at 65 °C. High stringency washes were carried out at 65 °C for 20 min. Membranes were washed twice in 2× SSC, 0.1% SDS. A final wash was performed in 0.25× SSC, 0.1% SDS.

#### Data acquisition

Images were captured by exposure to an imaging plate (Fuji) for 24 h, and spot intensities determined (MediaCybernetics Gel-Pro package).

### Oligonucleotide arrays

For the Affymetrix (Santa Clara, CA) HuGeneFL GeneChip (Hu6800, precursor of Human U95A GeneChip), reverse transcription, cDNA synthesis, labelling and data analysis were performed as described [22]. The default settings of the Affymetrix GeneChip 3.1 software were used to generate the average differences for this study. Publicly available oligonucleotide array data for ClonTech RNA applied to Affymetrix U95A GeneChips were downloaded for analysis from the Genomics Institute of the Novartis Research Foundation [16].

### cDNA microarrays — double-channel

#### Spotting

The microarrays were printed with a QArray (Genetix) instrument with 16 SMP2.5 pins (Telechem) on Ultra GAPS slides (Corning). The 3600 cDNA fragments were spotted in 50% DMSO in triplicate in three separate fields, in a 15 × 15 pattern within each block and with a feature centre-to-centre distance of 290 μm. The quality of the spotted slides was assessed by staining with Syto61 (Molecular Probes). The slides were UV cross-linked at 250 mJ/cm$^2$, followed by baking at 75 °C for 2 h, and post-processed with succinic anhydride/sodium borate solution.

#### *In vitro* transcription, labelling, and hybridization

The detailed protocols can be found on the web. For each single array experiment with distinguishable fluorescent dye labels for the individual RNAs, total RNA originating from one of the five tissues brain, heart, liver, lung and skeletal muscle was labelled during reverse transcription with either Cy3- or Cy5-labelled dUTP. A Universal Human Reference RNA (Stratagene) was labelled accordingly and used in all hybridizations.

### cDNA microarrays — single channel

#### Spotting

PCR products were purified with the QIAquick PCR Purification Kit (Qiagen), eluted with water, dried, and resuspended in 50% DMSO in water at a concentration of 100–200 ng/μl (as measured with an Agilent BioAnalyser). The products were spotted (417™ Arrayer, Affymetrix-GMS) onto CMT-GAPS™ amino silane coated slides (Corning) with 40–45% relative humidity at 22 °C. Samples were printed in triplicate. Slides were cross-linked (Stratalinker, Stratagene) with 65 mJ, followed by baking at 80 °C for 2 h.

#### Hybridization

Labelled cDNA was generated with the CyScribe First-Strand cDNA Labelling Kit (Amersham Pharmacia Biotech). 1 μg mRNA from each tissue was reverse transcribed in the presence of 'anchored' oligo(dT), random primer and Cy3-labelled dUTP, followed by degradation of RNA, neutralization and purification. The reverse-transcribed cDNA was mixed with 20 μg Cot-1 human DNA (Invitrogen), and mixed with 20 μg yeast tRNA (Invitrogen) and 20 μg pd(A)$_{40-60}$ (Amersham Pharmacia Biotech). Hybridizations were performed using labelled cDNA dissolved in a total volume of 25 μl 3.4× SSC, 0.3% SDS, at 65 °C for 15–18 h. After

Copyright © 2005 John Wiley & Sons, Ltd.

hybridization, the slides were washed at room temperature for 3 min each in 1× SSC, 0.03% SDS, 0.2× SSC, and 0.1× SSC. The slides were dried with $N_2$ gas and imaged with an Affymetrix 418 scanner (Affymetrix, Santa Clara, CA). Spot intensities were determined using the ArrayVision software package (Imaging Research Inc.).

### E-Northerns

Electronic Northern analysis [7] was based on the analysis of EST sequences annotated in the corresponding UniGene database record for each gene (**http://www.ncbi.nlm.nih.gov/UniGene/**).

### Data analysis

### ClonTech Northern blots

Band intensities for the target tissues were obtained from the Northern blot database. Unit vectors were created by dividing the band intensity for each tissue by the sum of all tissue values. In a few cases, there was no expression observed in the target tissues, and these vectors were defined as 'null' vectors. A portion of Northern blots displayed multiple bands (alternative transcripts). These were excluded unless the transcripts exhibited near-identical expression profiles (square root of sum of squares $< 0.15$). For those cases where expression was near-identical, the mean profile was used.

### RT-PCR

RT-PCR products were separated on agarose gels, an image captured, and the band intensities determined with the Gel-Pro software. For background correction, we subtracted the average empty lane value plus two standard deviations.

### Filter macroarrays

Intensity values from each hybridization (tissue) were normalized with reference to the median. Two distributions were apparent within the spot intensities for each filter (**http://www.cisreg.ca**). The distribution of lower values was judged to be consistent with background. Values were corrected for background by subtraction of the average of the background distribution plus two standard deviations.

### Oligonucleotide arrays

Calculations were based on the 'Average Difference Value' from the Affymetrix analysis software. For HuGeneFL GeneChips (Hu6800) and the Human U95A chips, average values were calculated for each tissue. Intensities were normalized by rescaling the entire data set in reference to a chosen baseline array. For both datasets, all values less than 20 were set to 20. Unit vectors were generated from the normalized data.

### cDNA microarrays — double-channel

Average intensities (with no background correction) of the triplicate spots were used for analysis. Background correction may reduce bias of ratios toward one, but at the cost of adding noise; here the variation in ratios was judged high enough, and the range of local background was low enough, that the decision was made to minimize noise. According to published procedures [23], for each array, a normalization factor $N$ was calculated by summing the measured intensities in both channels. In order to exclude the influence of extreme values, intensity values determined for the middle 66% of data points for each array were used to determine $N$. The data from one channel was scaled appropriately, and normalized expression ratios were transformed into logarithm base 2. All six arrays per tissue were averaged to obtain a single value per tissue per gene. Unit vectors were generated from the normalized and averaged data.

### cDNA microarrays — single-channel

Average intensities of the triplicate spots were used for analysis. In order to exclude extreme values, data were normalized to the average intensity values determined for the middle 66% of data points for each array. Unit vectors were generated from the normalized data.

### E-Northerns

Subsets of the cDNA libraries used for generation of ESTs in the global database were identified which corresponded to the five target tissues, and the number of ESTs derived from these libraries was determined for each gene. The libraries assigned to each tissue are indicated on the website (**http://www.cisreg.ca**). The raw EST

Copyright © 2005 John Wiley & Sons, Ltd.

counts were converted to percentages of the total number of ESTs produced from each library pool.

### Results

### Northern blot database — characteristics and format

Commercial multiple tissue Northern blots have been extensively used to profile expression of newly cloned genes. Two specific blots (MTN™, ClonTech, product numbers 7759-1 and 7760-1) dominate the scientific literature, each bearing RNA from eight tissues (7759-1: spleen, thymus, prostate, testis, ovary, small intestine, colon, peripheral blood leukocyte; 7760-1: heart, brain, placenta, lung, liver, skeletal muscle, kidney, pancreas). Image analysis was performed on a large collection of published Northern blots to generate a vector of relative abundances within each tissue for each transcript (defined by size). A total of 619 blots that addressed 535 distinct genes were analysed. Expression profiles for an average of 1.3 transcripts/gene were captured.

The dbMTN database containing the analysis results is available as an open-access resource for the public. A basic search engine is provided to enable researchers with their own multiple tissue Northern (MTN) results to search for human genes with similar expression profiles. dbMTN is available for downloading as a flat file consisting of 1398 tab-delimited rows, in which each row contains the profile for a transcript obtained with the indicated blot type. The data fields (columns) include transcript identifiers, GenBank accessions, GeneLynx accessions [15] (**http://www.genelynx.org**), bibliographic information, MTN blot type, and the relative abundance of the transcript across eight tissues. These 'scaled' values are provided, rather than raw band densities that cannot be compared between blots generated with probes of different intensities. Hyperlinks are provided to the original publications. The database and web interface are formatted to allow future acquisition of results from a new 12 tissue MTN product (product number 7780-1) that is gaining popularity.

Genes with uniform expression across diverse tissues can serve as valuable controls. Therefore, we identified genes with the most uniform expression across the 16 tissues represented on the two types of MTN blots. Four genes stood out as potentially appropriate loading controls for laboratory experiments: ACTB (actin, beta), AS3 (androgen-induced proliferation inhibitor), GAPD (glyceraldehyde-3-phosphate dehydrogenase), and GRB2 (growth factor receptor-bound protein 2). These genes were redundantly represented in the dbMTN collection and variation across the tissues was low for at least one transcript of each gene (data not shown). In addition to the transcript showing little variation across multiple tissues, ACTB and GAPD both produce highly expressed muscle-specific transcripts, which have not reduced their popularity as controls.

### Correlation analysis of MTN and microarray expression profiles

We compared expression profiles produced with ClonTech human RNA on multiple platforms. We generated profiles with HuGeneFL oligo arrays (Affymetrix, 7129 probes) and spotted cDNA microarrays (2608 probes), and incorporated external data for U95A oligo arrays (Affymetrix, 12600 probes). ClonTech RNA samples from brain, heart, liver and lung were used on all of the platforms. In order to measure the correlation between the large-scale microarray-generated profiles and the MTNs, we generated unit vectors for each gene's expression across the four tissues (as described in Methods). Correlation scores were calculated between the broadest possible intersections of genes for each pair-wise comparison (Table 1). Pearson correlation coefficients (PCCs) for pair-wise intersections of the three different microarray platforms, compared to Northern blots, were very similar and, overall, poor.

Given the diverse characteristics of the techniques and genes, different sub-groupings of the data can provide informative measures to identify potential strengths or weaknesses of the techniques. Genes were classified by the overall magnitude of expression based on total UniGene EST (expressed sequence tags) counts to reveal potential issues regarding sensitivity and/or dynamic range of the hybridization-based methods. When the data were classified according to the magnitude of expression, a performance difference could be observed between the cDNA microarrays and the two oligonucleotide arrays. For genes with low expression (low ESTs), results from the oligo

Copyright © 2005 John Wiley & Sons, Ltd. 

**Table 1.** Correlation coefficients reflecting similarity between the results obtained from microarray-based methods and Northern blots by analysing different levels of expression

| | Northern | cDNA array | Oligo array (Hu6800) |
|---|---|---|---|
| **All** | | | |
| **Northern** | — | | |
| **cDNA array** | 0.36 (93) | — | |
| **Oligo array (Hu6800)** | 0.42 (288) | 0.45 (1091) | — |
| **GNF oligo array (U95A)** | 0.35 (312) | 0.44 (1305) | 0.54 (2251) |
| **High EST** | | | |
| **Northern** | — | | |
| **cDNA array** | 0.45 (23) | — | |
| **Oligo array (Hu6800)** | 0.28 (72) | 0.55 (273) | — |
| **GNF oligo array (U95A)** | 0.29 (78) | 0.50 (326) | 0.56 (563) |
| **Middle EST** | | | |
| **Northern** | — | | |
| **cDNA array** | 0.35 (47) | — | |
| **Oligo array (Hu6800)** | 0.46 (144) | 0.45 (545) | — |
| **GNF oligo array (U95A)** | 0.36 (156) | 0.43 (653) | 0.55 (1125) |
| **Low EST** | | | |
| **Northern** | — | | |
| **cDNA array** | 0.26 (23) | — | |
| **Oligo array (Hu6800)** | 0.49 (72) | 0.33 (273) | — |
| **GNF oligo array (U95A)** | 0.40 (78) | 0.35 (326) | 0.52 (563) |

Pearson correlation coefficients were obtained in pair-wise comparisons of the relative expression levels between genes originating from the largest possible intersections between methods (number of genes considered in each comparison indicated in parentheses). Subsets of genes with different levels of expression were analysed according to the number of ESTs for each gene, (GNF = Genomics Institute of the Novartis Research Foundation). Additional figures are shown in the on-line supplementary material displaying scatter plots of the individual gene–gene correlations in each tissue and across all tissues.

arrays were better correlated with the MTN results, which may suggest superior sensitivity. At the high EST level, cDNA arrays performed slightly better, which points to potential quenching of the fluorescence signal for oligonucleotide arrays at high expression levels.

### Correlation analysis for a pre-selected set of genes — gene selection

In order to further explore the variation in performance for genes with different characteristics and to extend the analysis to other common methods including low-throughput approaches, we selected a set of 49 well-characterized human genes for subsequent analyses (gene IDs provided on website). The selection of these 49 genes was based on their presence both in the Northern blot database (dbMTN) and on the Affymetrix HuGeneFL oligonucleotide array. We focused on groups of genes representing different classes of expression based on the Northern blot results (blot type 7760-1) across five tissues targeted for laboratory analysis (heart, brain, lung, liver and skeletal muscle): *broad* (expression observed in at least three tissues), *selective* (expression in two tissues), *specific* (expression only in a single tissue) and 'null' (no expression detected in the target tissues on the 7760-1 MTN blot). Positions of the genes on the array were random and were not taken into consideration during the selection process or during subsequent profiling with other array-based methods.

### Expression profiles from high- and low-throughput techniques

Expression profiles were determined across the target tissues for the 49 selected genes. New profiles were produced for this report using ClonTech RNA via RT-PCR, filter macroarrays, single-channel and double-channel cDNA microarrays, and an oligonucleotide array (Affymetrix Hu6800). Published data were included in the analysis for oligonucleotide microarrays (GNF, Affymetrix U95A) [16] and 'Electronic Northerns' (dbEST), based on EST counts for each gene [17]. The U95A microarray results generated with ClonTech RNA were only available for four of the target tissues (heart, brain, lung and liver). While gene content was highly uniform, for some techniques individual genes were absent (e.g. three genes could not be amplified in the RT-PCR study with multiple primer pairs). The full datasets can be found on the project website.

After processing, data comprising all five tissues and the 49 genes were represented as unit vectors describing the relative pattern of expression across the target tissues (Figure 1). The expression profiles were split into the above-mentioned classes based on the breadth of gene expression in the Northern blots. Within the categories, genes were sorted by decreasing magnitude of expression based on total EST counts (i.e. from highest to lowest within each category).

Copyright © 2005 John Wiley & Sons, Ltd. 

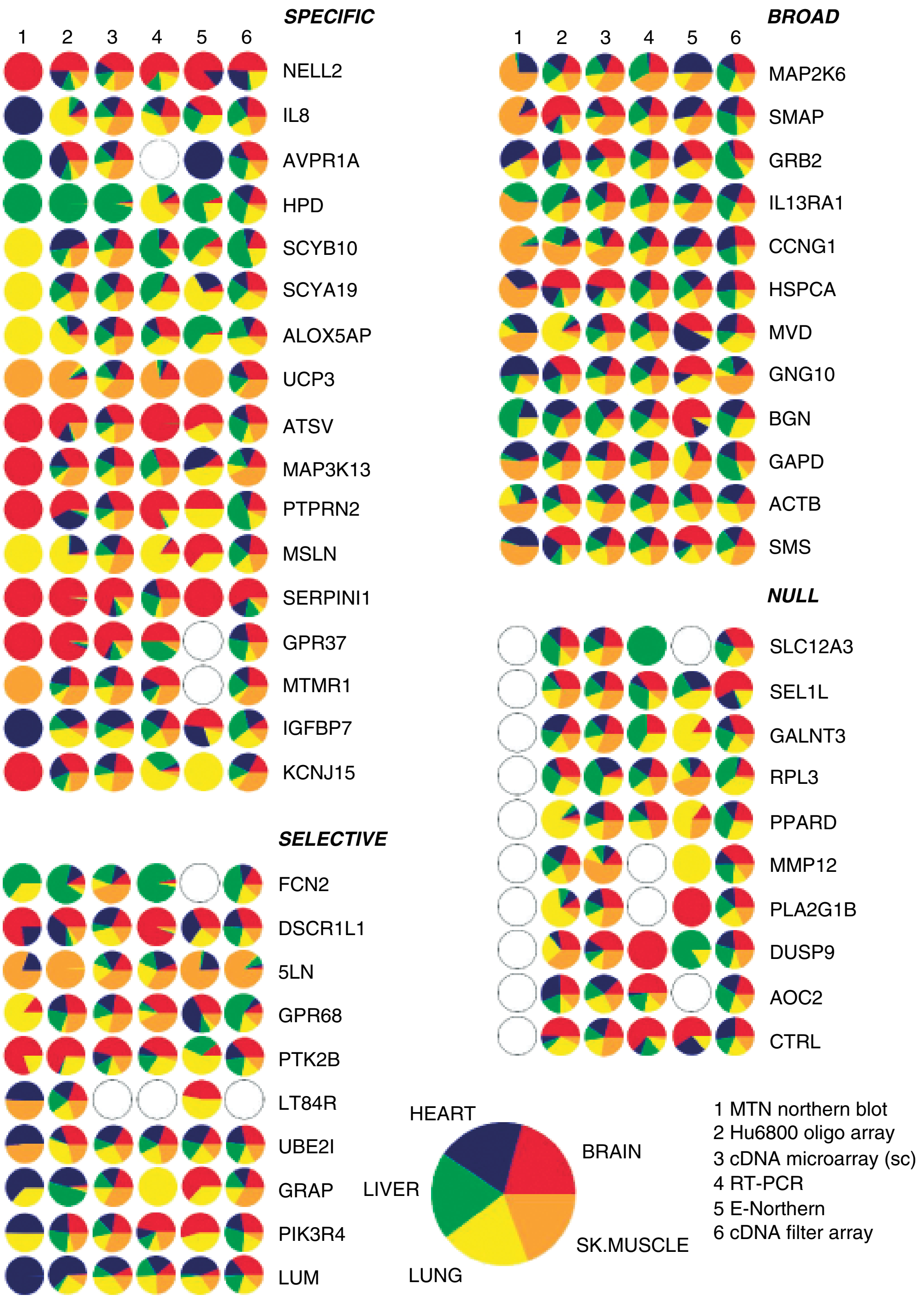

**Figure 1.** Relative expression levels for 49 genes in five tissues. Pie-charts are presented with the fraction of observed expression displayed for each of five target tissues. The genes are categorized based on the breadth of expression observed in published Northern blots across the five tissues analysed in this study. Within each category, the genes are ordered from highest magnitude of expression to the lowest, where the magnitude refers to the total number of EST sequences in dbEST for each gene. Each gene is identified by its official HUGO gene symbol (sc = single channel)

Copyright © 2005 John Wiley & Sons, Ltd. 

**Table 2.** Correlation coefficients reflecting similarity between the results obtained from different methods by analysing patterns of expression of a selected set of genes

| | Northern | Oligo array (Hu6800) | GNF oligo array (U95A) | cDNA array — dc | cDNA array — sc | RT-PCR | E-Northern |
|---|---|---|---|---|---|---|---|
| **All** | | | | | | | |
| **Northern** | — | | | | | | |
| **Oligo array (Hu6800)** | 0.50 (49) | — | | | | | |
| **GNF oligo array (U95A)** | 0.36 (39) | 0.51 (38) | — | | | | |
| **cDNA array — dc** | 0.61 (16) | 0.67 (16) | 0.56 (13) | — | | | |
| **cDNA array — sc** | 0.43 (48) | 0.57 (48) | 0.52 (38) | 0.77 (15) | — | | |
| **RT-PCR** | 0.37 (45) | 0.42 (45) | 0.31 (36) | 0.18 (14) | 0.22 (45) | — | |
| **E-Northern** | 0.25 (49) | 0.29 (49) | 0.38 (39) | 0.18 (16) | 0.21 (48) | 0.32 (45) | — |
| **Macro array** | 0.21 (48) | 0.39 (48) | 0.20 (38) | 0.38 (15) | 0.23 (48) | 0.26 (45) | 0.16 (48) |
| **Specific** | | | | | | | |
| **Northern** | — | | | | | | |
| **Oligo array (Hu6800)** | 0.65 (17) | — | | | | | |
| **GNF oligo array (U95A)** | 0.56 (12) | 0.71 (12) | — | | | | |
| **cDNA array — dc** | Na | Na | Na | — | | | |
| **cDNA array — sc** | 0.53 (17) | 0.77 (17) | 0.62 (12) | Na | — | | |
| **RT-PCR** | 0.51 (16) | 0.53 (16) | 0.35 (11) | Na | 0.26 (16) | — | |
| **E-Northern** | 0.40 (17) | 0.47 (17) | 0.58 (12) | Na | 0.40 (17) | 0.53 (16) | — |
| **Macro array** | 0.37 (17) | 0.44 (17) | 0.41 (12) | Na | 0.40 (17) | 0.37 (16) | 0.34 (17) |
| **Selective** | | | | | | | |
| **Northern** | — | | | | | | |
| **Oligo array (Hu6800)** | 0.57 (10) | — | | | | | |
| **GNF oligo array (U95A)** | 0.26 (10) | 0.49 (9) | — | | | | |
| **cDNA array — dc** | Na | Na | Na | — | | | |
| **cDNA array — sc** | 0.31 (9) | 0.49 (9) | 0.45 (9) | Na | — | | |
| **RT-PCR** | 0.40 (9) | 0.38 (9) | 0.48 (9) | Na | 0.16 (9) | — | |
| **E-Northern** | 0.16 (10) | 0.30 (10) | 0.20 (10) | Na | 0.25 (9) | 0.27 (9) | — |
| **Macro array** | 0.33 (9) | 0.58 (9) | 0.31 (9) | Na | 0.22 (9) | 0.23 (9) | 0.30 (9) |
| **Broad** | | | | | | | |
| **Northern** | — | | | | | | |
| **Oligo array (Hu6800)** | 0.18 (12) | — | | | | | |
| **GNF oligo array (U95A)** | −0.42 (9) | 0.22 (9) | — | | | | |
| **cDNA array — dc** | Na | Na | Na | — | | | |
| **cDNA array — sc** | 0.50 (12) | 0.40 (12) | 0.43 (9) | Na | — | | |
| **RT-PCR** | 0.34 (12) | 0.21 (12) | 0.03 (9) | Na | 0.53 (12) | — | |
| **E-Northern** | 0.02 (12) | −0.11 (12) | 0.26 (9) | Na | 0.04 (12) | −0.10 (12) | — |
| **Macro array** | −0.35 (12) | 0.12 (12) | −0.73 (9) | Na | 0.01 (12) | 0.19 (12) | −0.40 (12) |

Pearson correlation coefficients were obtained in comparisons of the relative expression levels between selected sets of genes. Number of genes considered in each comparison indicated in parentheses (dc = Double-channel; sc = single-channel; GNF = Genomics Institute of the Novartis Research Foundation).

### Correlation of expression data between techniques for selected gene set

In order to assess the similarity of the results obtained with different techniques, PCCs were calculated for every pair-wise comparison between techniques (Table 2). Similar correlation analyses were performed with Spearman Rank-Order coefficients (**http://www.cisreg.ca**). All of the statistical assessments led to qualitatively equivalent results.

For the entire set of genes, microarray-based expression profiling techniques and RT-PCR correlated best with Northern blots. When the data were categorized according to the pattern of expression on Northerns, a wide range of correlation scores were observed. The correlation was greatest for tissue-specific genes, with markedly lower correlation scores observed for selectively and broadly expressed genes (Table 2). Most genes judged to be accurately expressed (highest correlation with

Copyright © 2005 John Wiley & Sons, Ltd.

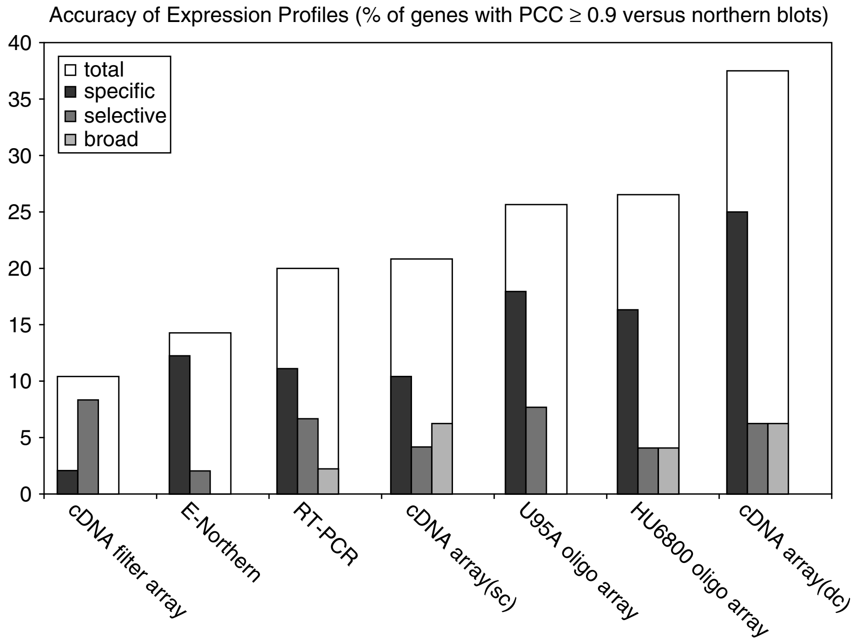

**Figure 2.** Accuracy of expression profiling. Bar-plot depicts percentage of accurately profiled genes defined by PCC $\geq$ 0.9 between the indicated method and Northern blots. Genes with restricted expression are prevalent (dc = double channel; sc = single channel). The inner bars delineate the fraction of the total contribution from genes falling into three classes of expression (specific to one tissue, selective expression in only two tissues, and broad)

Northern blot data) were tissue-specific (Figure 2). Both RT-PCR and single-channel microarrays displayed less variation across the expression categories. When the data were classified according to the magnitude of expression (based on EST counts/gene), the highest correlations were observed for genes with moderate expression levels (data available on project website).

### Discussion

As Northern blots have long served as a *de facto* standard for gene expression analysis in molecular biology, we created a literature-derived database of results produced with a specific commercial Northern blot to serve as a reference dataset. We performed a quantitative comparison of diverse expression profiling methods against the dbMTN data to identify techniques well suited for high-throughput analysis of human gene expression. Correlations of the results with the published data were consistently strongest for both cDNA and oligonucleotide microarrays. The cross-platform comparison provides a foundation for discussion and demonstrates the value of the MTN reference collection for the assessment of diverse approaches.

Creation of the dbMTN resource was dependent upon the extraction of image files from electronic publications. The preponderance of MTN-containing papers within three journals and the generous permission from the publishers to download the files were essential for the initial construction. Future expansion of dbMTN, and creation of similar resources, will be facilitated by the expansion of open-access policies for data in the scientific literature [18].

There are several possible explanations for the generally poor correlation observed between results from different platforms. One could argue that the correlation coefficients are misrepresenting the qualitative similarity of the data. This becomes particularly apparent during the analysis of broadly expressed genes, where the lowest correlations are observed. Pearson correlation coefficients might not be suited to compare quantitative readouts of a broad set of genes captured with diverse expression profiling techniques. To explore this possibility, a range of different concordance measures have been applied to assess the comparative performance of

Copyright © 2005 John Wiley & Sons, Ltd.

methods, all of which gave qualitatively similar results to those reported (Reimers, unpublished). For each platform, there are inherent characteristics that influence the results. The E-Northerns are limited by the available cDNA libraries, which were generated from diverse RNA samples and, in some cases, were prepared using normalization procedures to increase transcript diversity. RT-PCR is highly sensitive and has limited dynamic range, potentially over-representing the relative abundance of transcripts in tissues in which the gene is expressed at low levels. The variety of probes used in the different platform studies could also introduce inconsistencies. For genes with alternatively expressed transcripts, the different probes may hybridize to different subsets of the transcripts. The protocols were specifically selected to be consistent with standard laboratory practices, not necessarily to maximize correlation.

An important point to consider is the source of the RNA for cross-platform comparisons. In particular, we sought to maximize consistency of RNA samples. The Northern blots were produced with RNA pools generated with a defined preparation procedure by a single commercial provider (ClonTech). All of the RNA samples used in this study were obtained from ClonTech in order to minimize technical variability. The RNA samples are from pools of tissue obtained from multiple donors. We believe the focus on using RNA from a single source is an essential requirement to minimize variability.

The magnitude of transcript concentration in the RNA samples influences performance of profiling methods in different ways. Gene expression profiles from both oligonucleotide microarrays were most similar to the Northern results for genes with low transcript levels (Table 1). Sensitivity does not appear to be prohibitive. However, we recognize that genes available in the Northern blot database may be biased in favour of those with higher levels of expression. An alternative interpretation is that the methods perform worse for genes with high levels of expression, suggesting that some of the methods are impacted by saturated signals. The cDNA microarrays, on the other hand, performed best for genes expressed at higher levels.

The choice of a primary expression profiling technique is dependent upon each scientist's research topic and targeted set of genes. We conclude, based on the sets of genes used in this study, that oligonucleotide or cDNA microarrays are the preferred expression profiling techniques (among those examined) for the generation of data that is most consistent with the standard of traditional Northern blots. Microarrays are well-suited for comparisons of thousands of genes within two RNA samples, while PCR-based approaches may be preferable for in-depth analysis of a single gene across many samples. As the correlation scores observed between platforms are not encouraging, we believe that an emphasis on multi-technique validation of expression profiles is justified.

Several popular techniques were not addressed in this study, including spotted oligonucleotide arrays, quantitative PCR and SAGE. Quantitative PCR, which has become a preferred technique for gene-specific expression profiling, requires extensive optimization for each primer pair [11], and was judged to be cost-prohibitive in the scope of this study. SAGE analysis, a sequencing 'tag'-based method, offers access to significantly larger data pools than the EST-based electronic Northerns. While compatible SAGE libraries were not available for our comparisons, a recent study compared SAGE, E-Northerns and oligonucleotide arrays [19]. The study, which focused on individual tissues and selectively expressed genes, produced correlation scores in the same range as those we obtained for specifically expressed genes (Table 2). Recently, arrays of long oligonucleotides have emerged as a high-throughput option for expression profiling. Published results with long oligonucleotide arrays are highly correlated with results obtained using the Affymetrix platform [20]. The pace of innovation of expression profiling technologies continues to offer new methods for consideration.

The dbMTN collection is a valuable resource for researchers assessing the performance of expression profiling methods. In order to facilitate further exploration of the relative merits of diverse techniques and protocols, we have provided an extensive project website (**http://www.cisreg.ca**). dbMTN and the data produced in this study should provide fruitful opportunities to explore different analysis procedures, and we strongly encourage others to perform similar studies or apply their analysis procedures to the data we generated. To encourage others to make quantitative comparisons for specific laboratory or computational approaches, we will post relevant updates to the

Copyright © 2005 John Wiley & Sons, Ltd. 

website detailing alternative methods or interpretations.

### Acknowledgements

We are grateful for the advice and suggestions provided by Drs Harold Swerdlow, Zicai Liang, Anthony Brookes, and Tom Freeman. A comprehensive list of MTN publications was kindly provided by Kristi Dohner (ClonTech). We thank the publishers of Genomics, the Journal of Biological Chemistry and Proceedings of the National Academy of Sciences of the USA for image access. We thank Stephanie Wasserman for assistance with densitometry and gene annotation. We acknowledge Agilent and The Wallenberg Consortium North (WCN) for providing access to equipment and Pharmacia Corp. for their financial support to the Center for Genomics and Bioinformatics.

### References

1. Taniguchi M, Miura K, Iwao H, Yamanaka S. 2001. Quantitative assessment of DNA microarrays — comparison with Northern blot analyses. *Genomics* **71**: 34–39.
2. Ishii M, Aburatani H. 2000. Direct comparison of GeneChip and SAGE on the quantitative accuracy in transcript profiling analysis. *Genomics* **68**: 136–143.
3. Freeman WM, Vrana KE. 2000. Fundamentals of DNA hybridization arrays for gene expression analysis. *BioTechniques* **29**: 1042–1055.
4. Jordan BR. 1998. Large-scale expression measurement by hybridization methods: from high-density membranes to 'DNA Chips'. *J Biochem* **124**: 251–258.
5. Lander ES. 1999. Array of hope. *Nature Genet* **21**: (suppl): 3–4.
6. Ramdas L, Coombes KR, Baggerly K, Abruzzo L, Highsmith WE, *et al*. 2001. Sources of nonlinearity in cDNA microarray expression measurements. *Genome Biol* **2**: research 0047.1–0047.7.
7. Audic S, Claverie JM. 1997. The significance of digital gene expression profiles. *Genome Res* **7**: 986–995.
8. Madden SL, Landes G. 2000. Serial analysis of gene expression: from gene discovery to target identification. *DDT* **5**: 415–425.
9. Adams MD, Kerlavage AR, Fleischmann RD, Fuldner RA, Bult CJ, *et al*. 1995. Initial assessment of human gene diversity and expression patterns based upon 83 million nucleotides of cDNA sequence. *Nature* **377**: 3–174.
10. Freeman WM, Walker SJ, Vrana KE. 1999. Quantitative RT-PCR: pitfalls and potential. *Biotechniques* **26**: 112–122, 124–125.
11. Wang T, Brown MJ. 1999. mRNA quantification by real time TaqMan polymerase chain reaction: validation and comparison with RNase protection. *Anal Biochem* **269**: 198–201.
12. Kuo WP, Jenssen TK, Butte AJ, Ohno-Machado L, Kohane IS. 2002. Analysis of matched mRNA measurements from two different microarray technologies. *Bioinformatics* **18**: 405–412.
13. Gnatenko DV, Dunn JJ, McCorkle SR, Weissman D, Perrotta PL, *et al*. 2003. Transcript profiling of human platelets using microarray and serial analysis of gene expression. *Blood* **101**: 2285–2293.
14. Tan PK, Downey TJ, Spitznagel EL Jr, Xu P, Fu D, *et al*. 2003. Evaluation of gene expression measurements from commercial microarray platforms. *Nucleic Acids Res* **31**: 5676–5684.
15. Lenhard B, Hayes WS, Wasserman WW. 2001. GeneLynx: a gene-centric portal to the human genome. *Genome Res* **11**: 2151–2157.
16. Su AI, Cooke MP, Ching KA, Hakak Y, Walker JR, *et al*. 2002. Large-scale analysis of the human and mouse transcriptomes. *Proc Natl Acad Sci USA* **99**: 4465–4470.
17. Banfi S, Guffanti A, Borsani G. 1998. How to get the best of dbEST. *Trends Genet* **14**: 80–81.
18. Tamber PS, Godlee F, Newmark P. 2003. Open access to peer-reviewed research: making it happen. *Lancet* **362**: 1575–1577.
19. Huminiecki L, Lloyd AT, Wolfe KH. 2003. Congruence of tissue expression profiles from Gene Expression Atlas, SAGEmap and TissueInfo databases. *BMC Genom* **4**: 31.
20. Barczak A, Rodriguez MW, Hanspers K, Koth LL, Tai YC, *et al*. 2003. Spotted long oligonucleotide arrays for human gene expression analysis. *Genome Res* **13**: 1775–1785.
21. Podowski RM, Sonnhammer EL. 2001. MEDUSA: large scale automatic selection and visual assessment of PCR primer pairs. *Bioinformatics* **17**: 656–657.
22. Olsen MK, Roberds SL, Ellerbrock BR, Fleck TJ, McKinley DK, *et al*. 2001. Disease mechanisms revealed by transcription profiling in SOD1-G93A transgenic mouse spinal cord. *Ann Neurol* **50**: 730–740.
23. Quackenbush J. 2002. Microarray data normalization and transformation. *Nature Genet* **32**: 496–501.

Copyright © 2005 John Wiley & Sons, Ltd. 
